# Cholera Morbus

**Published:** 1876-08

**Authors:** 


CHOLERA MORBUS.—Dr. Burrows says: In attacks of ordinary cholera morbus, the best remedy that I have found is composed of equal parts of the tinctures of rhei, capsicum, camphor, opium, and spearmint, of which thirty drops in a little water are to be taken every hour until the disease is checked. In cholera infantum I have had better success with the following than with any remedy I have used: Syr. rhei, 2 ounces; aqua calces, 4 drachms; and aqua menth. pip., 2 drachms. Of this give to a child a year old a teaspoonful every hour until it operates. The object is to change the character of the discharges. If they are devoid of bile, give ten drops of the fluid ext. leptandria, in a little syrup, every two or three hours, until they change odor. Did I know who the originator of the above formula was, I would cheerfully give him credit; but as I do not, I can only bear testimony to its excellence, by actual experience in its use.—*The Medical Brief.*

DEATH FROM ETHER.—Dr. E. L. Holmes, of Chicago, reports (*Chicago Med. Journ. and Examiner*, May, 1876) a case of death following the administration of ether, for the extraction of cataract.—*Medical News and Library.*

Ergot in Chronic Uterine Catarrh.—As the operation of ergot given internally is very tedious, and its hypodermic injection is attended with difficulties in private practice, Dr. Goreleitschenko has tried the effect of penciling the canal of the cervix uteri with solution of ergot (ergotin, 2 scruples; glycerin and distilled water, of each 2 drachms), leaving the pencil within the canal from one to three minutes. In this way he has treated twenty-nine cases of chronic metritis attended with chronic catarrh. In twenty-two of these discharge ceased, the swollen state of the mucous membrane disappearing, and the uterus itself being smaller and firmer. The subjective symptoms also ceased, and regular menstruation ensued. In the other seven cases the improvement which ensued was soon followed by relapse, the uterus in these cases having undergone more advanced alterations.—*St. Petersburg Medical Woch.*

Liberal Government.—In addition to the Government grant of £1000 per annum, made to the Royal Society to enable that body to defray the expenses of scientific investigations considered by a committee of the society to be worthy of such aid, it has been proposed by her Majesty's Government to grant an increase of £4000, leaving the conditions under which the original sum is administered undisturbed—*Med. Times and Gaz.*

Skulls—Ancient and Modern.—Prof. Rolleston (*Brit. Med. Jour.*, Sept. 4, 1875,) says the largest result to which craniometry and cubage of skulls have attained is, to my thinking, the demonstration of the following facts, viz: First, that the cubical contents of many skulls, from the earliest sepultures from which we have any skulls at all, are considerably larger than the average cubical contents of modern European skulls; and secondly, that the female skulls of those times did not contrast to the disadvantage with the skulls of their male cotemporaries which the average female skulls of modern days do, when subjected to a similar comparison.—*Detroit Review of Medicine.*

Poisoning from Quinine.—Dr. J. J. Putnam has reported a case in which, when two or three grains of quinine were administered, swelling of the lips and tongue, with redness of the face and neck, was produced.

